# An Innovative Minimally Invasive Oncoplastic Technique for Early Breast Cancer: The Spoon-Shape Technique

**DOI:** 10.3390/jcm11051379

**Published:** 2022-03-02

**Authors:** Zhilin Chen, Xuefeng Shi, Wenjie Shi, Zihao Chen, Jiajia Zeng, Jie Dong, Rui Zhuo, Rudy Leon De Wilde

**Affiliations:** 1 Department of Breast Surgery, Hainan Medical University, Haikou 570102, China; zhilinchen@hainmc.edu.cn; 2 University Hospital for Gynecology, Pius-Hospital, University Medicine Oldenburg, 26121 Oldenburg, Germany; wenjie.shi@gltcmac.org.cn; 3 Department of Breast Surgery, Guangxi TCM University, Guilin 541002, China; xuefeng.shi@gltcmac.org.cn (X.S.); zihao.chen@gltcmac.org.cn (Z.C.); jiajia.zeng@gltcmac.org.cn (J.Z.); jie.dong@gltcmac.org.cn (J.D.); 4 Department of Breast Surgery, EUSOMA Certified Breast Center No.1037/00, Guilin 541002, China

**Keywords:** oncoplastic, breast cancer, breast-conserving surgery, spoon-shape technique

## Abstract

Here, we describe a step-by-step novel level I oncoplastic technique and present the aesthetic results of 58 breast cancer patients who underwent the spoon-shape technique for primary tumor resection. The Paris Breast Center’s 5-point scale was used to evaluate the aesthetic outcomes. The median age of the participants was 52 years old. The average size of the resected tumor was 22.1 mm; two intraoperative re-excisions were required due to positive margins. Postoperative localized seroma was observed in four patients, and one patient presented signs of wound infection. Skin flap necrosis and fat liquefaction were not observed. The average aesthetic score was 4.86. None of the patients presented cancer recurrence in the following two years. The spoon-shape technique showed good aesthetic results because it provided the surgeons an adequate amount of surrounding tissue from which to reshape the breast after tumor removal. We encourage surgeons to apply this approach in early-stage breast cancer, regardless of the quadrant where the tumor is located.

## 1. Introduction

Breast-conserving surgery (BCS) combined with postoperative radiotherapy has been proven to have the same good clinical prognosis as mastectomy for early-stage breast cancer [1,2]. However, there are still some issues that need to be solved, such as a high positive margin rate and unsatisfactory aesthetic results, especially in cases with small-sized breasts, large-sized tumors, or with special tumor localization [3,4]. Oncoplastic approaches could be considered as an alternative to mastectomy to complete tumor removal while also assuring satisfactory aesthetic results [5,6].

Clough B developed a system atlas for breast oncoplastic surgery (OPS), classifying OPS approaches as level I and level II, according to the tumor localization, tumor volume, and breast reshaping necessity. When the excised volume is less than 20% of the breast volume and no mammoplasty reshaping is needed, the level I techniques are applied, which include glandular flaps that can be advanced or rotated to fill the cavity. For larger resections, between 20 and 50% of the gland, mammoplasty techniques, which fall under level II OPS, are required [7,8]. Previous studies have shown that these approaches are safe and provide good esthetic results. However, level I techniques have two main concerns that need to be resolved: the first is how to design an approach that could be applied to different breast quadrants, and the second is how to obtain sufficient surrounding health tissue to reshape small-sized volume breasts while obtaining satisfactory aesthetic results.

In our breast cancer centers, we are committed to the development of new OPS approaches to solve these matters. In our preliminary report [9], we described another innovative level II OPS technique, the Zhuo technique, for patients with small- to medium-volume breasts. This approach uses flaps from the inframammary fold to provide sufficient glandular tissue to reshape the breast after the removal of lower-inner quadrant tumors. In the present report, we describe a novel level I oncoplastic technique, the spoon-shape technique, which can be applied to tumors located in any breast quadrant.

## 2. Materials and Methods

We retrospectively analyzed 58 cases of patients who underwent surgery using the spoon-shape technique between January 2018 and December 2019 in the Department of Breast surgery, Guangxi TCM University, China. The Paris Breast Center’s 5-point scale was used to evaluate the aesthetic outcomes [9,10]. Descriptive statistical methods were used to present the outcomes. Approval from the Ethics Committee of EUSOMA Certified Breast Center No.1037 (GTCMH-2020-100; 1 September 2020) was obtained.

### 2.1. The Spoon-Shape Approach Design

**Figure 1:**
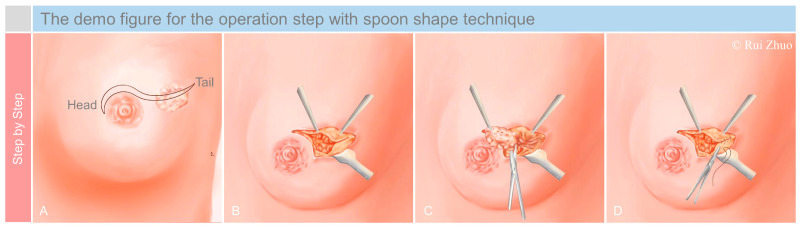
Step-by-step demo figure of the spoon-shape technique. (**A**) Approach design; (**B**) exposure of the surgical area; (**C**) tumor removal; (**D**) defect repair.

Patient who has previously been confirmed to have early breast cancer is placed in a flat position to perform a new ultrasound examination to locate the tumor; thereafter, a circular mark delineating the tumor is painted on the skin (Figure 1). Patients then provide consent to undergo a sentinel lymph biopsy and, in accordance with the results, to further axillary dissection.

The initial incision is performed in an arc shape on the diameter line of the marked circle, facing the areola, and it is extended to the areolar rim, creating a final shape resembling a spoon (Figure 1A). A second spoon-shaped incision is made from the areolar rim to the tumor area, and the resulting flap skin is removed. As observed, we refer to the incision surrounding the mammary areola as the “head” and the incision across the tumor as the “tail” parts of the incision.

### 2.2. Tumor Removal

The spoon-shaped incision provides adequate surgical access to the tumor. It should be assured that the edges of the excised surgical specimen are more than one centimeter from the tumor margins.

The excised tissue should be analyzed to confirm that it is free of tumor cell margins. If the margins are negative, the next step is performed; otherwise, the surgeon needs to re-excise the tumor until the pathology report shows negative margins (Figure 1B,C). When the tumor is completely excised, a residual cavity is left, and we consider filling it with the glandular tissue surrounding the defect.

### 2.3. Defect Repair

The assistant uses hooks to expose the surgical area while the surgeon squeezes the breast tissue around the residual cavity to assess the volume of the defect.

If the defect is small, we only need to use the tail of the incision for the gland to be rearranged. If the defect is larger, we need to free the gland around the residual cavity to separate the whole gland tissue from the pectoralis major muscle. On the other hand, the tissue in the spoon technique is removed, resulting in a gap between the superior and inferior edges of the incision. This gap could allow the head of the spoon incision and around tissues to become more flexible. So, next, we just need to rotate the free gland on one side along the areola to fill the postoperative defect.

Finally, the glandular tissue is sutured, and the skin of the breast surface is aesthetically closed.

### 2.4. Aesthetic Evaluation

The aesthetic intraoperative evaluation is completed by the surgeon and the assistant. The breast shape, the position of the nipple–areola complex, and the symmetry of the bilateral breasts are evaluated. The postoperative aesthetic evaluation is carried out one year after the operation by means of the 5-point scale proposed by the Paris Breast Center [9]. In addition, patient self-satisfaction is also important; therefore, we regularly register patient satisfaction during postoperative follow-up.

## 3. Results

### 3.1. Baseline Characteristic of Patients

A total of fifty-eight patients underwent the spoon technique. The baseline characteristics of the cases are shown in Table 1. The median age of the patients was 52 years old (range from 33 to 79 years). The median BMI was 23.9 kg/m^2^ (range from 18.4 to 31.8 kg/m^2^). Half of all of the patients had medium-sized breasts (A cup 24.5%; B cup 53.4%; C cup 12.1%). The median tumor size was 22.1 mm (range 12 to 45 mm), and most of them were identified as having invasive ductal carcinoma (IDC) (94.8%, 55 cases) that was mostly of the Luminal B subtype (44.8%; 26 cases). Triple-negative breast cancer (TNBC) was identified in 3.4% of cases (2/58).

### 3.2. Surgical Outcomes

The mean operation time was 35.2 min (range 30 to 45 min), excluding SLN and rapid-freezing waiting time. No patients presented lymph-node metastasis. The average bleeding volume was 27.1 mL (range from 20 to 35 mL). Two patients underwent two re-excision procedures to obtain negative margins. Four patients presented minor postoperative complications. Seroma was observed in three of them, which was resolved with needle aspiration (*n* = 2) and a compression bandage (*n* = 1). The other patient presented with localized skin redness and swelling, which resolved with antibiotic treatment. Skin flap necrosis and fat liquefaction were not observed.

### 3.3. Aesthetic Outcome

**Figure 2:**
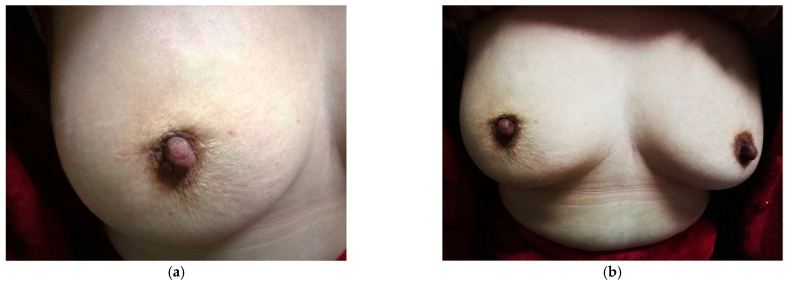
The aesthetic outcomes of a patient a who underwent the oncoplastic spoon-shape technique. (**a**) The breast has a natural shape and a fine scar. (**b**) The symmetry of bilateral breasts after surgery.

**Figure 3:**
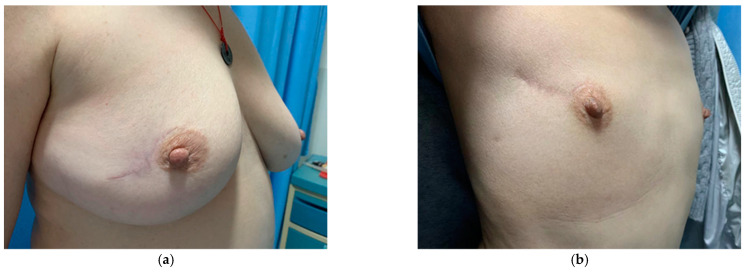
Apply spoon-shape technique to different volume size breast with tumor account for larger percentage. The shape and superficial scare of patient’s breast with large volume (**a**) and small volume (**b**) after tumor removed.

The aesthetic outcomes were evaluated by at least three breast surgeons. The average score was 4.86, with most of the patients scoring 5 points (*n* = 52), and six scoring 4 points. No recurrence occurred within two years of follow-up. Figure 2 shows the aesthetic outcomes of a patient at the one-year follow-up. As observed, the breast has a natural shape, the scar is concealed within the areolar rim (Figure 2a), and the breast is symmetrical to the contralateral breast (Figure 2b). As shown in Figure 3, we found that this technique is also favorable for breasts with larger defects after tumor removal, no matter the volume size. In addition, Patient self-evaluation results indicate that all follow-up patients were satisfied with the outcome of this procedure.

## 4. Discussion

Oncoplastic surgical approaches have helped to expand the indications for breast-conserving therapies, allowing mastectomy to be avoided. The level I techniques are especially easy to apply in routine clinical practice and are able to be performed to obtain complete primary tumor excision while conserving the breast shape [10]. Actually, breast and plastic surgeons work together to improve OPS techniques, allowing patients to achieve better aesthetic results [11,12]. Based on this collaborative work, we developed a new level I technique, called the spoon-shape technique, for patients with early breast cancer located in any quadrant of the breast.

It is known that the distribution of glandular tissues varies in different breast quadrants; therefore, different surgical incisions have been proposed according to tumor localization. The batwing incision is a common approach when a tumor is located in the upper-inner quadrant, where two circular incisions as well as de-epithelialization are performed, and the nipple–areola complex (NAC) is relocated, which has the possibility of shifting the NAC to one side or of changing the symmetry of the breast [13]. In contrast, the incision we designed can reduce these risks because de-epithelialization is not required, and the nipple can be adjusted during the operation by remodeling the “head part” of the spoon-shaped incision.

The fusiform incision is mainly used for tumors in the upper-outer quadrant. This design follows the natural skin folds, and the two incisions are tapered at the corners, resulting in a wound with low tension that, in turn, facilitates healing. However, skin retractions could occur if the defect is not well filled, or great asymmetry with the contralateral breast could occur in cases of peri-areolar tumors [14]. In contrast, the “tail part” of the spoon-shaped incision can be adjusted in terms of both length and width according to the tumor location and tumor size, facilitating the advancement of healthy tissue to reconstruct the breast.

A fish-hook-shaped incision would be a better choice for tumors in the lower-inner quadrant and is more applicable in patients with large-sized breasts rather than in patients with small- to medium-sized breasts [11]. This technique usually leaves a long scar on the breast surface, affecting the cosmetic appearance of the breast. The J incision is used for tumors in the lower-outer quadrant and leaves bird-beak-like deformities on the breast surface [10]. In our technique, the length of the incision generally ranges from 1 to 5 cm depending on the size of the breast; with an average of 1.5~2 cm, the scar length is very short. In addition, since the tissues in the spoon technique are removed, resulting in a gap between the superior and inferior edges of the incision. This gap could allow the head of the spoon incision and around tissues to become more flexible, so we just rotate the tissue away from the NAC, not closing the side. After the two edges have been aligned, the arc-fashion incision could avoid the occurrence of skin retractions or bird-beak-like deformities.

The round block technique is widely promoted as a classic breast-conserving procedure, and so many patients could benefit from this plastic surgery [15]. When we compare our technique with this surgery procedure, we find that round block is not favorable for tumor away from the areola while spoon technique adds an additional tail pointing to the tumor location and can easily expose the operation area. In addition, the spoon technique just needs to rotate the gland part; a minimally invasive incision to repair the defect means less fat necrosis. However, round block would use a bigger incision, freeing more glands, which entails a high risk of fat necrosis, although it brings a larger surgical field of view.

Severe surgical complications can delay adjuvant treatment in patients, thereby increasing the risk of disease recurrence [16]. Contrary to other reports, in our study, only four transitory minor surgical-related complications were observed [17]. In addition, no fat necrosis was recorded in our study, we infer that the possible reason is that our technique involves rotation of the tissue, including the skin and glands, and no extensive freeing of the glands. Moreover, no recurrence or metastasis was observed within the next two years after surgery. These results indicate that our technique is safe and effective. In our study, we observed that the arcs on both edges of the incision can be adjusted based on the tumor location, facilitating the advancement of gland tissue to cover the defect and decreasing the risk of scar retraction or asymmetry. A total of 87.9% of patients had A- or B-cup-sized breasts, and the average postoperative aesthetic score was 4.86 points, showing that our technique was able to achieve satisfactory aesthetic results when applied to patients with small- to medium-volume breasts presenting with early-stage breast cancer.

Although our technique could provide a favorable level I OPS technique for breast cancer patients, there are still some limitations that cannot be ignored. First, when the tumor location is far away from the nipple, we need to design a longer tail incision, which increases the length of the postoperative scar. In addition, long-term follow-up to evaluate the oncology outcome is also necessary.

## 5. Conclusions

The use of new oncoplastic approaches could increase the aesthetic and oncological outcomes of breast-conservative therapies. In our analysis, we found that our level 1 OPS, the spoon-shape technique, can be used to perform a complete lumpectomy of tumors of different sizes and localizations. Additionally, this approach allows surgeons to have an adequate amount of surrounding tissue to fill the defect after tumor removal, resulting in a cosmetic scar and the breast having a natural shape. We encourage surgeons to apply this novel technique in early-stage breast cancer, regardless of the quadrant where the tumor is located. Further studies are needed to evaluate the oncology outcomes, with long-term follow-up, of the spoon-shape technique.

## Figures and Tables

**Table 1:** Baseline characteristics of patients who underwent spoon-shape technique.

Characteristics	Overall (*n* = 58)	
	Number	%
Age (Years)		
≤60	39	67.2
>60	19	32.8
BMI ^a^ (kg/m^2^)		
18–25	41	70.7
>25	17	29.3
Cup (%)		
A	20	34.5
B	31	53.4
C	7	12.1
Tumor Size (mm)		
12–20	26	44.8
20–45	32	55.2
Histology (%)		
IDC ^b^	55	94.9
ILC ^c^	1	1.7
Other ^d^	2	3.4
Subtype (%)		
Luminal A	19	32.8
Luminal B	26	44.8
HER2	11	19.0
TNBC ^e^	2	3.4
Location (%)		
Left	38	65.5
Right	20	34.5

^a^ Body mass index; ^b^ invasive ductal carcinoma; ^c^ invasive lobular carcinoma; ^d^ including mucinous adenocarcinoma and metaplastic breast carcinoma; ^e^ triple-negative breast cancer.

## Data Availability

The datasets presented in this study can be obtained from the corresponding author.
